# The effect and mechanism of volatile oil emulsion from leaves of *Clausena lansium* (Lour.) Skeels on *Staphylococcus aureus in vitro*

**DOI:** 10.3389/fmicb.2024.1376819

**Published:** 2024-03-08

**Authors:** Yan-Na Guo, Ke-Ren He, Shao-Shan Liang, Rui-Wei Mou, Meng-Han Lu, Yong-Ming He, Lu-Ping Tang

**Affiliations:** ^1^School of Life Science and Engineering, Foshan University, Foshan, China; ^2^Department of Biomedical Sciences, City University of Hong Kong, Hong Kong, China

**Keywords:** leaves of *Clausena lansium* (Lour.) Skeels, volatile oil, emulsion, *Staphylococus aureus*, *Salmonella typhimurium*

## Abstract

This study aimed to develop a suitable dosage form of volatile oil from wampee leaves and to explore its antibacterial mechanism *in vitro*. The chemical composition of the volatile oil from wampee leaves was determined by gas chromatography-mass spectrometry (GC-MS). Different microemulsion ratios were tested and their stabilities were investigated to determine the optimal ratio. The minimum inhibitory concentration (MIC) and minimum bactericidal concentration (MBC) of the wampee leaves volatile oil emulsion (WVOE) against *Salmonella typhimurium* (*S. typhimurium*) and *Staphylococcus aureus* (*S. aureus*) were determined using double-dilution and plate-counting methods, respectively. Morphological changes in these two bacteria were observed using scanning electron microscopy. Death, ultrastructural morphology, and biofilm formation were also assessed for *S. aureus*. Finally, we established an *S. aureus*-infected Lewis lung carcinoma (LLC) cell model to evaluate the protective effects of the volatile oil emulsion and the associated mechanisms. The volatile oil extracted from wampee leaves contained 37 compounds, of which 96.49% were aromatic hydrocarbons, terpenoids, and their oxygen-containing derivatives. The emulsion was most stable at 1:1 in the oil phase and 1:9 in the water phase. WVOE had poor antibacterial activity against *S. typhimurium*, but the MIC and MBC against *S. aureus* were 312.5 and 2,500 μg/mL, respectively. *S. aureus* survival rates were 84.6%, 14.5%, and 12.8% in the 1/2, 1, and 4 × MIC groups, respectively, compared with 97.2% in the control group. *S. typhimurium* survival was not affected by WVOE treatment. WVOE administration induced cavity formation and abnormal binary fission, and significantly inhibited biofilm formation in *S. aureus* cells. The WVOE notably reduced the number of *S. aureus* and inhibited *TLR4*, *NLRP3*, *NF-κB*, *IL-6*, *IL-18*, and *TNF-α* gene expression in *S. aureus*-infected LLC cells. The WVOE had a significant inhibitory effect on *S. aureus* and altered its cell membrane permeability. Moreover, it alleviated inflammation by inhibiting the NF-κB-NLRP3 pathway in *S. aureus*-infected LLC cells.

## Graphical Abstract

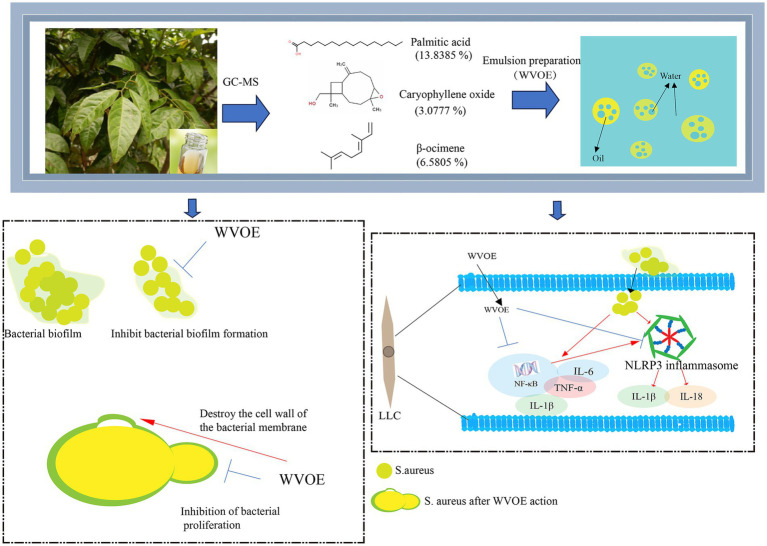

## 1 Introduction

*Staphylococcus aureus* is a common gram-positive bacterium widely present in the environment. It can enter the body through the respiratory and digestive systems and surface wounds, resulting in pneumonia, enteritis, and bacteraemia (Ding et al., 2011; Zheng et al., 2017). According to a previous study, *S. aureus* can colonise the nasal cavity in humans, and 30% of such infections are permanent. *S. aureus* is also the main cause of skin infections. In more severe cases, it enters deeper tissues through the skin, causing bacteraemia (Wertheim et al., 2005; Dryden, 2009; Tong et al., 2015). Currently, death due to *S. aureus* infection occurs at a rate of approximately 20%, and it is strenuous to reduce mortality further (Asgeirsson et al., 2018). *S. aureus* infection can cause arthritis and dermatitis in poultry and exudative dermatitis, suppurative mastitis, skin abscesses, and pneumonia in livestock, causing serious economic losses to the livestock and poultry industries (Graveland et al., 2011; Ribeiro et al., 2018; Haag et al., 2019). It is also highly resistant to antimicrobial drugs. Therefore, its treatment is not ideal.

There have been many advances in the treatment of infectious diseases using traditional Chinese medicine (TCM), even for most drug-resistant bacteria. Volatile oils used in TCMs contain various bioactive substances that have antibacterial (Chen et al., 2019; Yu et al., 2019; Zhang et al., 2022), anti-inflammatory (Chen et al., 2018; Lyu et al., 2019; Ting et al., 2021), and antioxidant activities. Previous studies have shown that the volatile oils of *Atractylodes* can inhibit *Helicobacter pylori* in a dose-dependent manner by inhibiting biofilm growth and bacterial reproduction (Yu et al., 2019). Volatile oil from the roots of *Atractylodes* is effective against *S. aureus*, *S. albicans*, and *Bacillus subtilis* (Zhang et al., 2022). Linalool, terpene, alcohol, terpene, ene, and methyl palmitate in the volatile oil of *C. aurantii* exert synergistic bacteriostatic effects (Zhang et al., 2020). The volatile oil of rattan pepper has an inhibitory effect on *Escherichia coli*, *Shigella dysenteriae*, and *S. aureus*, and can inhibit bacterial cells and destroy the bacterial cell membrane and cell wall (Cheng et al., 2022). Thus, the volatile oils used in TCM are widely used in the field of biomedicine and are one of the research hotspots for the reduction of veterinary antibacterial drug use in China.

*Clausena lansium* (Lor.) Skeels (wampee) are plants of the Rutaceae family that are widely cultivated in the Lingnan region of China and have a long medicinal history (Huang et al., 2023). The seedless wampee is a rare fruit unique to Lingnan County in Guangdong Province. It has been awarded honorary titles such as “Geographical Indication Protection Product” and “Chinese Famous Fruit.” According to the literature, wampees are pungent and bitter (Xiao, 2009). They function as an antipyretic and in tonifying qi, resolving phlegm, and detoxifying, and are mainly used to treat fever, malaria, cough, phlegm, and asthma (Zhao, 2009). The rich aroma of wampee leaves is related to their volatile oil content. Modern pharmacological studies have shown that wampee leaves have antibacterial (Jia et al., 2017), cough-relieving, asthma-relieving, and liver-protecting effects (Li et al., 2012; Huang et al., 2015). However, whether the essential oil from wampee leaves have bacteriostatic effects and its associated mechanisms still not yet been clarified. Therefore, in order to promote the development and utilization of wampee leaves, and improve it’s medicinal value, the bacteriostatic action and mechanism of wampee leaves were studied.

Volatile oils have the advantages of requiring a small dosage and having a significant effect, with high safety. However, their volatility and rich taste make them not conducive to preservation or use in animal feed. Therefore, there is an urgent need to prepare a dosage form to protect their stability and mask their strong taste. Emulsion is a homogeneous liquid preparation method that can be used to mix incompatible liquids. It has the advantages of high dispersity, rapid absorption, and high bioavailability (Taleb et al., 2018). Making emulsions from volatile oils can ensure accurate drug dosage, whereas oil-in-water emulsions can mask the taste of the volatile oil itself, making it easier for animals to consume (Hasssanzadeh et al., 2018). Therefore, in this study, the volatile oil from wampee leaves was converted into an emulsion, providing the basis for subsequent experiments.

In summary, the aim of this study was to prepare a volatile oil emulsion of wampee leaves (WVOE) and explore its antibacterial effects and mechanisms *in vitro*, so as to develop a TCM drug for the prevention and treatment of *S. aureus* infection.

## 2 Materials and methods

### 2.1 Bacteria and cells

*S. typhimurium* (ATCC 14028) and *S. aureus* (BNCC186335) were provided by Beina Biotechnology Co., Ltd. (Guangzhou, China), and Lewis lung carcinoma cells (LLCs, TCM-C742) were provided by Haixing Biotechnology Co., Ltd. (Fujian, China).

### 2.2 Extraction and analysis of volatile oil from wampee leaves

Wampee leaf powder was obtained using a 100-mesh sieve. Fifty grams of the powder was then soaked in 500 mL of distilled water for 2 h and in steam for 4 h to obtain the volatile oil. Gas chromatography-mass spectrometry (7890A-5975C; Agilent Technologies, Santa Clara, CA, United States) was used to determine the chemical composition of the volatile oil.

### 2.3 Preparation of volatile oil emulsion from wampee leaves

Different proportions of volatile oil and emulsifier (Tween 80) were mixed to form an oil phase. The oil phase droplets were added to water at 80°C and stirred with a magnetic stirrer (500 rpm) to make sure the total volume of volatile oil emulsion was 2 mL (Table 1). A stability experiment was then conducted on the volatile oil emulsions of wampee leaves. The microemulsion was stored in a refrigerator at 4°C for 1 month, and its appearance changes were observed. The emulsion was also placed in an incubator at 50 ± 2°C for 5 days and observed for precipitation and layering. The optimal microemulsion ratio was selected based on the experimental results.

**Table 1:** Composition design of volatile oil emulsion from wampee leaves.

Experiment number	Volatile oil content (mg)	Emulsifier content (μL)	Water content (μL)
1	100	100	1,800
2	100	200	1,700
3	100	300	1,600
4	100	400	1,500
5	100	500	1,400
6	100	600	1,300
7	100	700	1,200
8	100	800	1,100
9	100	900	1,000

After selecting the optimal ratio, we conducted a diluted stability experiment. One hundred microlitres of the emulsion was added to 10 mL of standard hard water, and after stirring constantly for 30 s, the emulsion was immediately transferred to a clean, dry 10 mL measuring cylinder. The cylinder was placed in a water bath for 1 h at a constant temperature of 30°C, and was observed.

### 2.4 The antibacterial effect of the volatile oil emulsion from wampee leaves on *Salmonella typhimurium* and *Staphylococcus aureus*

#### 2.4.1 Preparation of bacterial suspension

Single colonies were inoculated into 15 mL of Luria-Bertani (LB) broth and cultured on a shaker for 8 h. One millilitre of the bacterial solution was centrifuged at 5,000 rpm for 10 min, and the bacteria were collected. They were washed twice with sterile phosphate-buffered saline (PBS) and resuspended in sterile PBS to obtain a bacterial suspension of 10^9^ CFU/mL.

#### 2.4.2 Determination of minimum inhibitory and bactericidal concentrations against *Salmonella typhimurium* and *Staphylococcus aureus*

The minimum inhibitory concentration (MIC) was determined using the double-dilution method. First, the emulsion was diluted to different concentrations with liquid LB culture medium, and then the control, Tween 80, and positive control groups were treated with different drug concentrations. Ten millilitres of liquid LB culture medium was added to each test tube (the above groups), followed by 0.1 mL of a 10^6^ CFU/mL bacterial solution. After 12 h of cultivation at 37°C, the growth of the bacteria was observed, and the optical density at 600 nm (OD_600_) before and after bacterial growth was determined. The MIC was determined when no bacterial growth was visible to the naked eye, and the OD_600_ value was significantly lower than the OD_600_ value of the positive control group. The minimum bactericidal concentration (MBC) was determined using the plate-counting method. One hundred microlitres of culture medium was taken from sterile test tubes with growth visible to the naked eye and applied to the plates. The plates were incubated at 37°C for 12 h, and the minimum concentration required for bacterial colony growth less than 5 was taken as the MBC.

#### 2.4.3 Time-antibacterial curve

One hundred microlitres of 10^5^ CFU/mL *S. typhimurium* and *S. aureus* suspensions were added to LB broth containing different concentrations of the emulsions. The cells were then cultured at 37°C for 0, 2, 4, 8, 12, 24, 36, and 48 h, and the quantity of bacteria produced in the time mentioned above period was determined. Finally, time-inhibition curves were plotted.

#### 2.4.4 Scanning electron microscopy

*S. typhimurium* and *S. aureus* were incubated in culture medium with drug concentrations of 2,500 μg/mL for 24 h. The bacterial cultures were centrifuged, collected, fixed, and observed under a scanning electron microscope (MC1000; HITACHI, Tokyo, Japan).

#### 2.4.5 Observation of *Staphylococcus aureus* death by flow cytometry after volatile oil emulsion treatment

*S. aureus* and *S. typhimurium* were incubated with 1/2, 1, and 4 × the MIC of the emulsion for 24 h. The bacterial cells were then centrifuged, and 1 mL of PBS was added before resuspension. Bacterial death was observed using fluorescein isothiocyanate and propidium iodide dyes, which determine whether a cell is dead by the intensity of the fluorescence that stains the cell membranes and nuclei, respectively. The number of bacteria was determined using flow cytometry (CytoFLEX; Beckman Coulter, Brea, CA, United States).

#### 2.4.6 Transmission electron microscopy

*S. aureus* was incubated with the emulsion at the MIC and MBC for 6 and 12 h, respectively. The effect of the emulsion on *S. aureus* was observed using a transmission electron microscope (HT7700, HITACHI, Tokyo, Japan).

#### 2.4.7 Detection of *Staphylococcus aureus* biofilm formation

Two hundred microlitres of different concentrations of the volatile oil emulsion medium containing *S. aureus* (10^8^ CFU/mL) were added to a 96-well plate, each concentration plated six times. The plate was then incubated a 37°C for 24 h and washed 3 times with PBS. The cells were fixed with 200 μL of methanol, after which, the methanol was removed and the cells were dried. They were then stained with a 1% crystal violet solution for 20 min, washed three times with PBS, and dried. Finally, 200 μL of 95% ethanol was added for 5 min and the OD_570_ was measured.

### 2.5 Establishment of *Staphylococcus aureus*-infected LLC cell model and drug treatment

#### 2.5.1 *Staphylococcus aureus* infection model in LLC cells

When the LLC cell density reached 70%, 2 mL of antibiotic-free culture medium and 100 μL of a 10^8^ CFU/mL *S. aureus* suspension were added to the culture medium. After 1, 1.5, and 2 h of bacteria exposure, the morphology of the LLC cells were observed under a microscope (Mshot, Guangzhou, China). *S. aureus* counts were determined after cell lysis. Furthermore, quantitative reverse transcription-polymerase chain reaction (qRT-PCR) was used to determine the gene expression levels of *NF-κB*, *TNF-β*, and *IL-1β* to verify that the suitable infection model.

#### 2.5.2 Drug toxicity evaluation

Toxicity experiments were divided into the following seven groups: control group, Tween group (emulsifier group), and different drug concentration groups (250, 125, 62.5, 31.25, and 15.62 μg/mL). The control group was supplemented with cell culture medium, the Tween group with 125 μL/mL Tween 80, and the drug groups were administered different concentrations of WVOE. After 24 h, 10 μL of Cell Counting Kit-8 (CCK-8) solution was added, and the cells were incubated for 1 h. The OD was measured at 450 nm using a microplate reader (iMark; Bio-Rad, Hercules, CA, United States). The experimental results were based on OD value. The higher OD value, the higher cell activity and cell proliferation, and the lower cytotoxicity of the drug.

#### 2.5.3 *Staphylococcus aureus* counts in LLC cells

This experiment was divided into the following six groups: control group, model group, Tween group (emulsifier group), high-dose drug group (62.50 μg/mL WVOE), medium-dose drug group (31.25 μg/mL WVOE), and low-dose drug group (15.62 μg/mL WVOE). Sixty-two and five tenths μL/mL Tween 80 was added to culture medium in Tween 80 group, and different WVOE concentrations were added to the drug groups. After 23 h of cultivation, 100 μL of 10^8^ CFU/mL of *S. aureus* was added to each group except the control group, and the cells were cultured for 1 h. The LLC cells were washed twice with PBS, and 200 μL of the cell lysate was added into LLC cells. After 10 min of reaction, 800 μL of PBS was added to stop cell lysis and the samples were mixed and diluted 10^5^-fold with sterile water, then 100 μL of the mixture was added to LB agar medium. Each group had three replicates, and the cells were incubated at 37°C for 24 h. The number of bacteria present was recorded.

#### 2.5.4 qRT-PCR analysis of inflammatory cytokines in LLC cells treated with WVOE

This experiment was divided into four groups: control, model, high-dose drug (31.25 μg/mL WVOE), and low-dose drug (15.62 μg/mL WVOE) groups. Twenty-three hours after drug administration to the LLC cells, 10^7^ CFUs of *S. aureus* was added to the cells and cultured for 1 h. Total RNA was extracted from the cells and reverse-transcribed into cDNA. qRT-PCR was performed to determine the gene expression levels of *NLRP3*, *IL-18*, *IL-6*, *TNF-α*, *TLR4*, and *NF-κB P65* in the LLC cells. The sequences of primers used for real-time PCR analysis were shown in Table 2.

**Table 2:** The sequences of primers used for real-time PCR analysis.

Gene	Forward primer (3′ to 5′)	Reverse primer (5′ to 3′)
IL-18	CAAAGTGCCAGTGAACCCCAGAC	ACAGAGAGGGTCACAGCCAGTC
IL-6	CTTCTTGGGACTGATGCTGGTGAC	TCTGTTGGGAGTGGTATCCTCTGTG
NF-κB	AGACCCAGGAGTGTTCACAGACC	GTCACCAGCCGAGTTATAGCTTCAG
NLRP3	CCCAGACCTCCAAGACCACTACG	CATCCGCAGCCAGTGAACAGAG
TLR4	GCCATCATTATGAGTGCCAATT	AGGGATAAGAACGCTGAGAATT
TNF-α	CGCTCTTCTGTCTACTGAACTTCGG	GTGGTTTGTGAGTGTGAGGGTCTG
β-actin	CTACCTCATGAAGATCCTGACC	CACAGCTTCTCTTTGATGTCAC

### 2.6 Statistical analysis

Quantitative data are expressed as the mean ± standard deviation. All statistical analyses and chart drawings were performed using GraphPad Prism 8 (GraphPad, San Diego, CA, United States). Differences between groups were analysed using a one-way non-parametric analysis of variance (ANOVA). Statistical significance was set at *p* < 0.05.

## 3 Results

### 3.1 Identification of chemical components in volatile oil extracted from wampee leaves by GC-MS analysis

**Figure 1:**
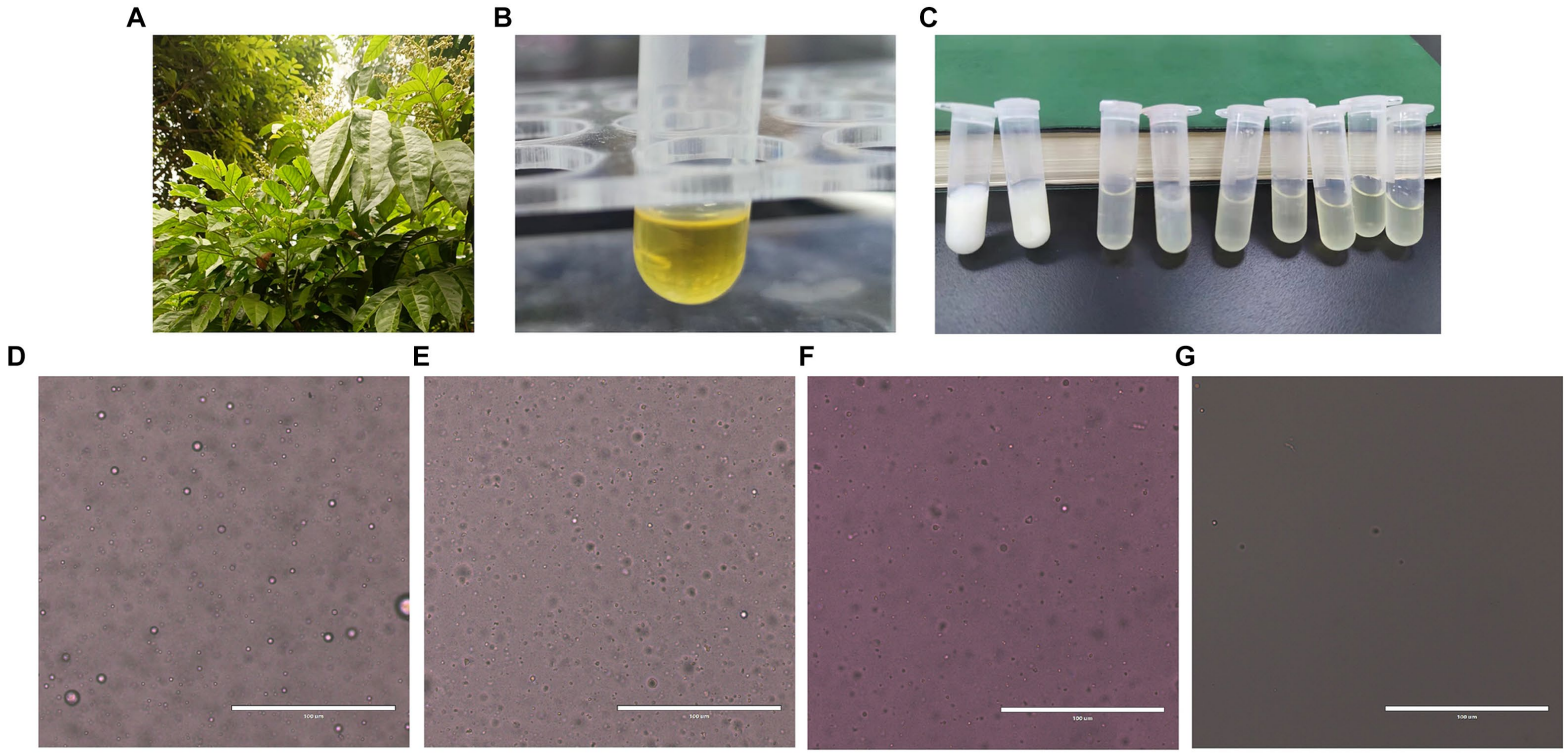
Preparation and stability test of WVOE. **(A)** Leaves of *Lausena lansium*. (Lour.) Skeels (wampee). **(B)** Wampee leaves volatile oil. **(C)** Preparation of WVOE by different ratio of emulsifier and water. **(D–G)** Microscopic observation of WVOE from stability testing (The scale is 100 μm). **(D)** 1 h after emulsion configuration. **(E)** Low temperature (4°C) for 1 month. **(F)** High temperature (50 ± 2°C) for 5 days. **(G)** GB/T 1603-2001 stability experiment.

According to the optimized extraction method, the volatile oil extracted from wampee leaves (Figure 1A) is golden in color and has a strong aroma (Figure 1B). Ethyl acetate extracted volatile oil and samples were analyzed by GC-MS to obtain a total ion flow spectrum, and calculated the relative content of each component using peak area normalization method. A total of 37 compounds were identified, mainly aromatic hydrocarbons, terpenoids, and their oxygen-containing derivatives, accounting for 96.49% of the total volatile oil. Twenty-four chemical ingredients from them were listed in Table 3. The main components of volatile oil were 5,5-dimethyl-1-vinylbicyclohexane (13.0509%), palmitic acid (13.8385%), β-Basilene (6.5805%), phytol (5.7725%), α-Red myrrh alcohol (2.8441%), caryophyllene oxide (3.0777%), eucalyptol alcohol (1.7751%), etc. Among them, the content of 5,5-dimethyl-1-vinylbicyclohexane and palmitic acid is relatively high.

**Table 3:** The chemical composition of volatile oil from wampee leaves.

Order	Retention time (min)	Molecular formula	Chemical compound	Relative content (%)
1	5.7159	C_12_H_22_	Benzene,1,3-bis(1,1-dimethylethyl)-	1.4325
2	7.2827	C_15_H_24_	Caryophyllene	0.9157
3	7.9805	C_14_H_22_O	2.4-Di-tert-butylphenol	0.4809
4	8.0811	C_15_H_24_	(−)-β-sesquisellene	0.6429
5	8.6036	C_15_H_24_O	Cyclohexene 3-(1,5-dimethyl-4-hexeny1-6methylene)-.[S-(R*.S*)]-	1.7751
6	8.6586	C_15_H_24_O	[1H-Cycloprople]azulen-7-oldecahydro-1.1.7-trimethyl-4-methylene-,[1ar-(1a.alpha.4a.alpha.7.beta.,7a.beta,7b.alpha.)]-	3.0777
7	8.9386	C_15_H_26_O	(1R.4R)-1-methyl-4-(6-Methylhept-5-en-2-y1)eyclohex-2-one	0.7376
8	9.3131	C_20_H_34_O	Thunbergol	0.5854
9	9.3652	C_15_H_26_O	.alpha.-Bisabolol	2.8441
10	10.1247	C_10_H_14_	1.3.8-p-Menthatriene	3.5833
12	10.4131	C_9_H_12_O_2_	2(3H)-Benzofuranone 3a,4.7.7a-tetrahydro-3a-methyl	1.2095
13	10.4602	C_15_H_22_O	2.6,1 1-Dodecat rienal.	1.0722
14	10.5258	C_15_H_24_	Bicyclo3.1.1hept-2-ene2,6-dimethyl-6-(4-methyl-3-pontenyl)-	1.0071
15	10.8208	C_15_H_24_	Trans-,alpha.-Bergamotene	2.5147
16	10.9899	C_10_H_16_	Camphene	2.6716
17	11.1299	C_10_H_16_	1,3,6-Octatriene,3,7-dimethyl-, (Z)	6.5805
18	11.1889	C_10_H_16_	5,5-Dimethyl-1-vinylbicyelo [2,1.1]hexane	13.0509
19	11.2285	C_16_H_32_O_2_	n-Hexadecanoic acid	13.8385
20	11.2655	CHO_4_	Dibutyl phthalate	3.3802
21	12.1696	C_20_H_40_O	Phytol	5.7725
22	12.3649	C_18_H_30_O_2_	Linoelaidic acid	2.6966
23	12.4658	C_18_H_36_O_2_	Octadecanoic acid	2.5675
24	15.9858	C_30_H_50_	Squalene	1.2833

### 3.2 Preparation and stability testing of volatile oil emulsion from wampee leaves

Wampee leaf Volatile oil emulsions were prepared using different emulsifier-to-water ratios. Based on the stability test, the volume ratio of wampee leaf volatile oil to emulsion was 1:1, and the oil phase (wampee leaf volatile oil emulsion, WVOE) was determined to be 1:9 in the aqueous phase (Figure 1C). Based on the selected optimal ratio, the diluted stability experiment results showed that the WVOE was milky white with a droplet size of less than 10 μm, and there was no precipitation, stratification, or flocculation under high-temperature conditions (Figures 1D–G).

### 3.3 Effects of WVOE on the bacteriostasis of *Salmonella typhimurium* and *Staphylococcus aureus*

**Figure 2:**
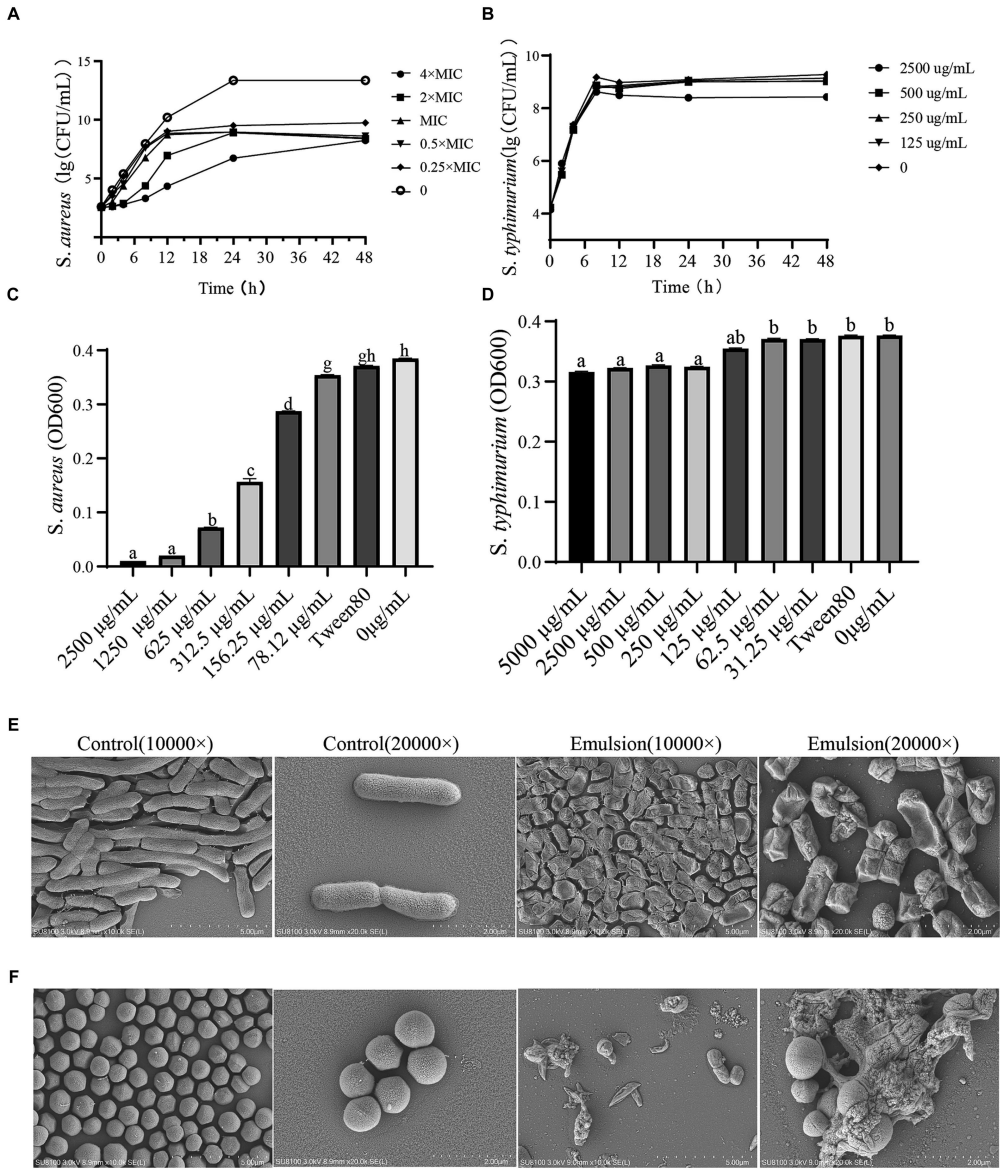
The effects of WVOE on bacteriostasis of *S. typhimurium* and *S. aureus*. Time-antibacterial curve of **(A)** *S. aureus* and **(B)** *S. typhimurium*. The difference of OD_600_ of **(C)** *S. aureus* and **(D)** *S. typhimurium*. The morphological changes of **(E)** *S. typhimurium* and **(F)** *S. aureus* after MBC of WVOE treated for 24 h by scanning electron microscopy.

The turbidity of the bacterial suspensions was observed and differences in optical density (OD) were determined. We found that the WVOE had no effect against *S. typhimurium*, while the MIC against *S. aureus* was 312.5 μg/mL and the MBC was 2,500 μg/mL (Figures 2C,D).

The WVOE had a weak antibacterial effect on *S. typhimurium*, with few wrinkles on the bacterial surface, but no sign of death (Figures 2B,E). However, it had a significant inhibitory effect on *S. aureus*, including the cell lysis and death of *S. aureus* after treatment with different concentrations of WVOE for 24 h (Figures 2A,F). By comparing the antibacterial effects of the WVOE on gram-positive (*S. aureus*) and gram-negative (*S. typhimurium*) bacteria, we found that the WVOE had a more significant antibacterial effect on gram-positive bacteria. Therefore, we further explored the antibacterial of the emulsion against gram-positive bacteria.

### 3.4 Effect of WVOE on bacterial death of *Staphylococcus aureus* and *Salmonella typhimurium*

**Figure 3:**
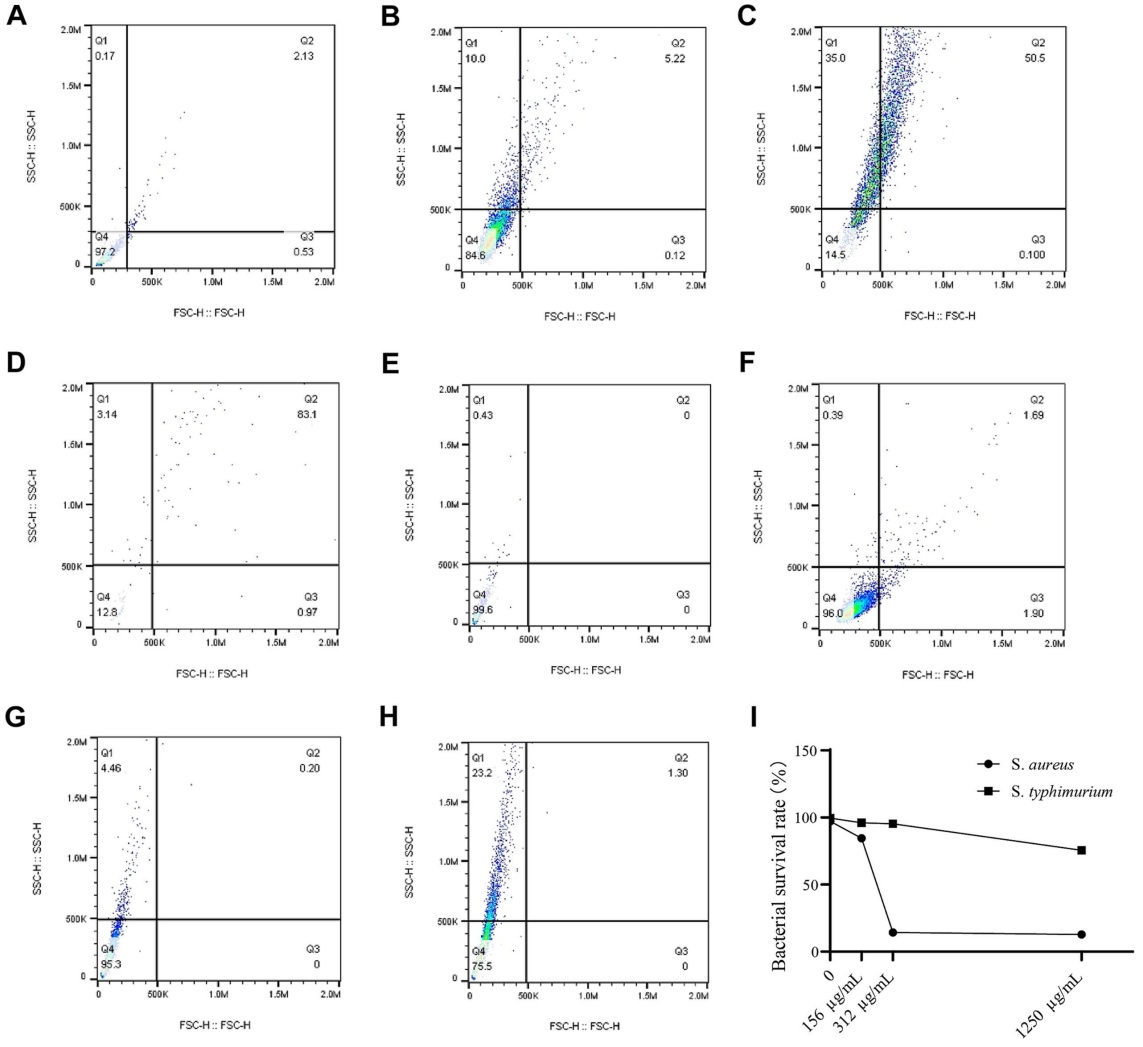
*S. aureus* and *S. typhimurium* survival rate after WVOE treatment was observed by flow cytometry. **(A)** Control group of *S. aureus*. **(B–D)** 1/2 × MIC, 1 × MIC and 4 × MIC group of *S. aureus*. **(E)** Control group of *S. typhimurium*. **(F–H)** 1/2 × MIC, 1 × MIC and 4 × MIC group of *S. typhimurium*. **(I)** Survival curve of *S. aureus* and *S. typhimurium*.

The bacterial death of *S. aureus* and *S. typhimurium* after different concentrations of WVOE for 24 h were measured using flow cytometry (Figure 3). Compared with control group, the *S. aureus* survival rates of 1/2 × MIC group, 1 × MIC group and 4 × MIC group were 84.6%, 14.5%, and 12.8%, respectively, which were significantly lower than those of the control group (97.2%). *S. aureus* have already lysed and died after 24 h WVOE treatment, so the number and can be stained of bacteria in 4 × MIC group decreased significantly. The *S. typhimurium* survival rates in 1/2 × MIC group, 1 × MIC group and 4 × MIC group were 96, 95.3, and 75.5%, respectively, which were similar to control group (99.6%). The results proved that the WVOE had a better antibacterial effect against *S. aureus*, and the antibacterial effect was dose-dependent.

### 3.5 Ultrastructural morphological changes of *Staphylococcus aureus* after WVOE treatment

**Figure 4:**
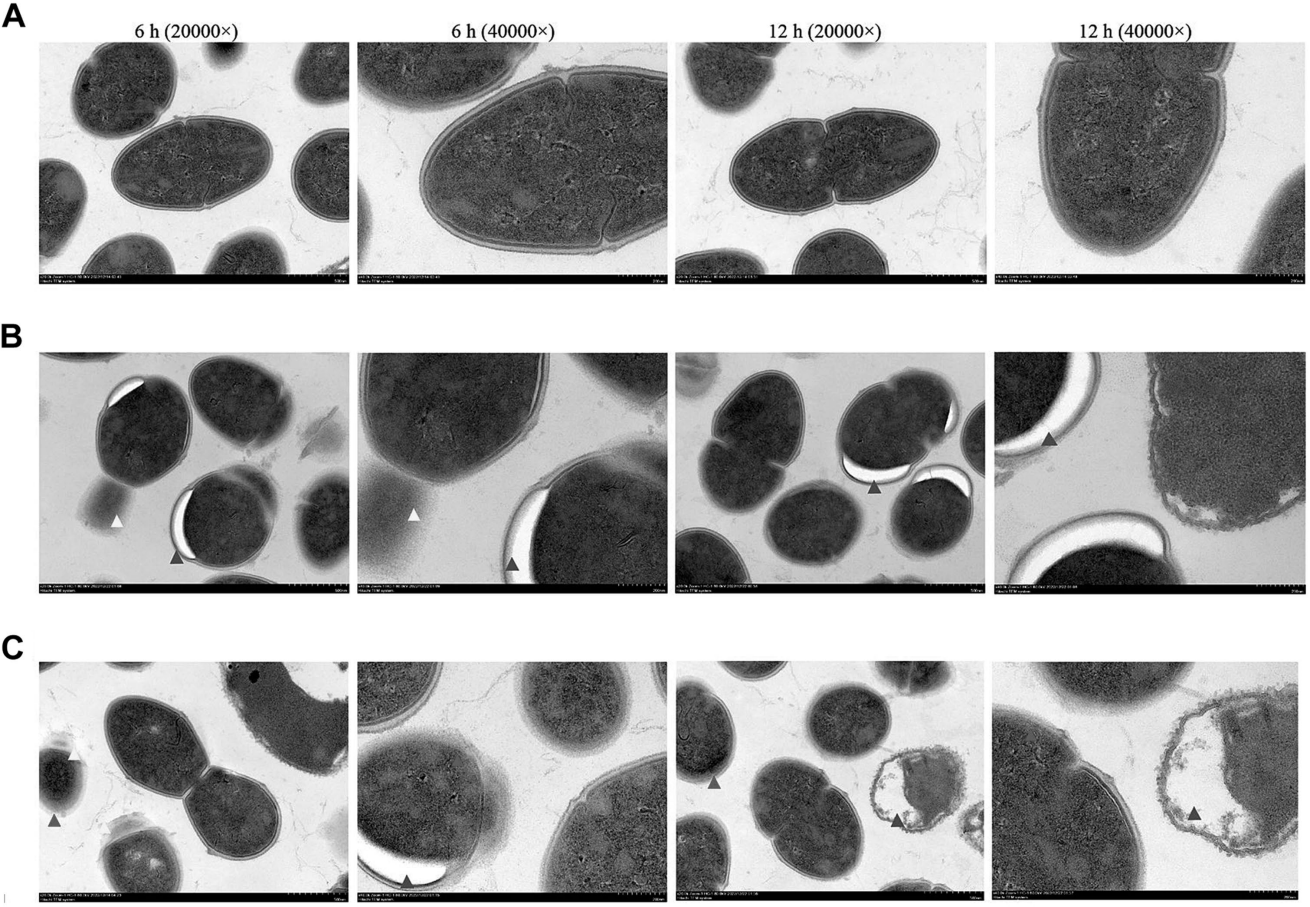
The ultrastructural morphology of *S. aureus* by transmission electron microscopy. *S. aureus* were observed after WVOE treated for 6 and 12 h. **(A)** Control group, **(B)** MIC group and **(C)** MBC group. Red arrows indicate blurred cell wall of the bacterial; blue arrows indicate the bacterial cavity; yellow arrows indicate inhibited bacterial division.

There was a clear demarcation between the cell membrane and wall of *S. aureus* in the control group. Some bacteria were undergoing binary fission, and their division was expected, with cells on both sides of the diaphragm being symmetrical (Figure 4A). After 6 h of treatment with the emulsion at its MIC, cavity formation and abnormal binary fission of the bacteria were observed, including incomplete and asymmetrical division. After 12 h, the cavity size gradually increased (Figure 4B). After treatment with the emulsion for 6 h at the MBC, bacterial binary fission was severely inhibited, seemingly without a membrane-like structure, and was accompanied by the formation of cavities. After 12 h, cell membrane and cell wall spacing within the bacteria disappeared and cell cavitation and death occurred (Figure 4C).

### 3.6 Effect of WVOE on *Staphylococcus aureus* biofilm formation

**Figure 5:**
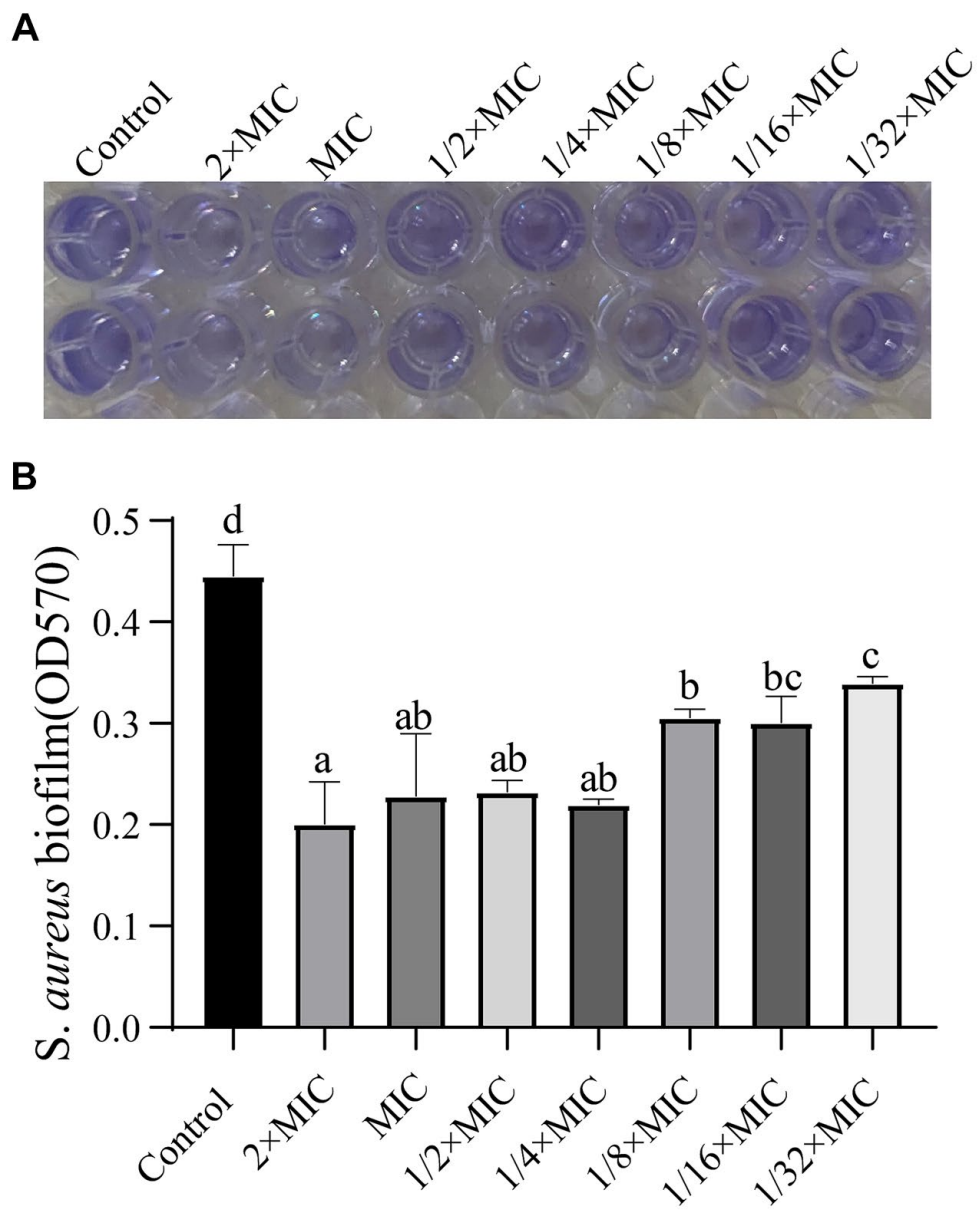
Effects of WVOE on *S. aureus* biofilm formation. Two hundred microlitres of different concentrations of WVOE were added into *S. aureus* and incubated at 37°C for 24 h. The biofilm formation of *S. aureus* was detected. **(A)** OD570 color rendering results, the darker the color, the more bacterial biofilm formation. **(B)** Bacterial biofilm growth under different drug concentrations. Different letters of shoulder label indicated statistical significance (*p* < 0.05). The same letter of shoulder label indicated no statistical significance (*p* > 0.05). The following image is the same as it.

As shown in Figure 5, the volatile oil emulsion significantly inhibited the formation of *S. aureus* biofilms (*p <* 0.05), and the inhibitory effect remained obvious at a 1/32 dilution of the MIC.

### 3.7 Establishing a cell model of *Staphylococcus aureus* infection

**Figure 6:**
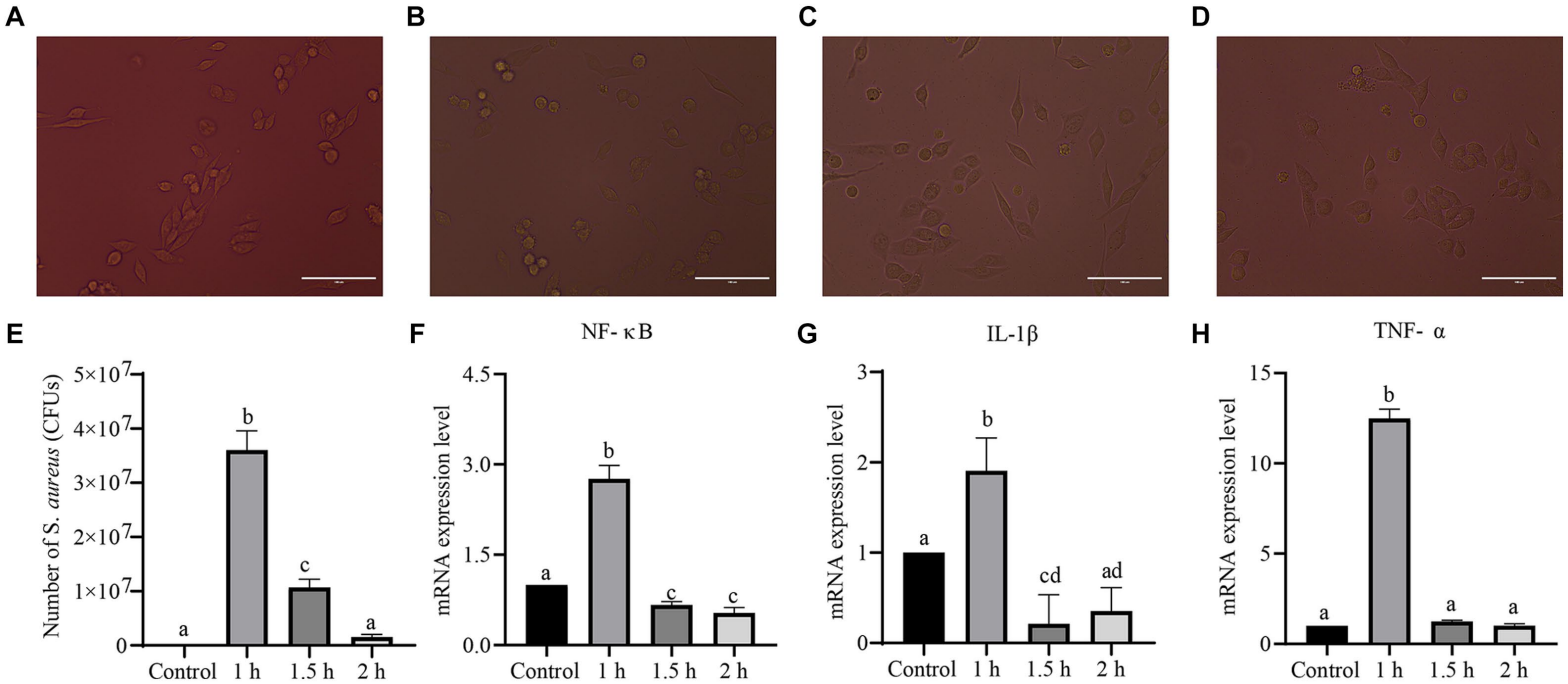
Establishment of a *S. aureus*-infected cell model. **(A–D)** Morphological changes of LLC cells infected with *S. aureus* for 0, 1, 1.5 and 2 h. **(E)** The bacterial load of LLC cells infected with *S. aureus*. **(F–H)** Genes levels of inflammatory factors (NF-κB, IL-1β, TNF-α) in LLC cells infected with *S. aureus*.

Cell morphology, the number of infected cells, and inflammatory factor levels were evaluated to investigate the infection of LLC cells with *S. aureus* (Figure 6). After 1 h of *S. aureus* infection, cell morphology changes were not significant, but the number of infected cells was the highest (*p <* 0.05) and the levels of inflammatory factors (*TNF-α*, *NF-κB*, and *IL-1β*) significantly increased (*p <* 0.05). After 1.5 h, cell morphology changed from irregularly elongated to elliptical, and cell death occurred. The number of infected cells and the levels of inflammatory factors decreased. After 2 h, some cells exhibited circular floating structures and died. These results indicated that, as the duration of bacterial infection increased, there was more cell death, resulting in a significant decrease in the bacterial count and inflammatory factor gene expression levels compared to 1 h of infection. Therefore, 1 h of *S. aureus* infection of LLC cells was chosen as the modeling time.

### 3.8 The antibacterial effects and mechanisms of action of the volatile oil emulsion on *Staphylococcus aureus*-infected LLC cells

**Figure 7:**
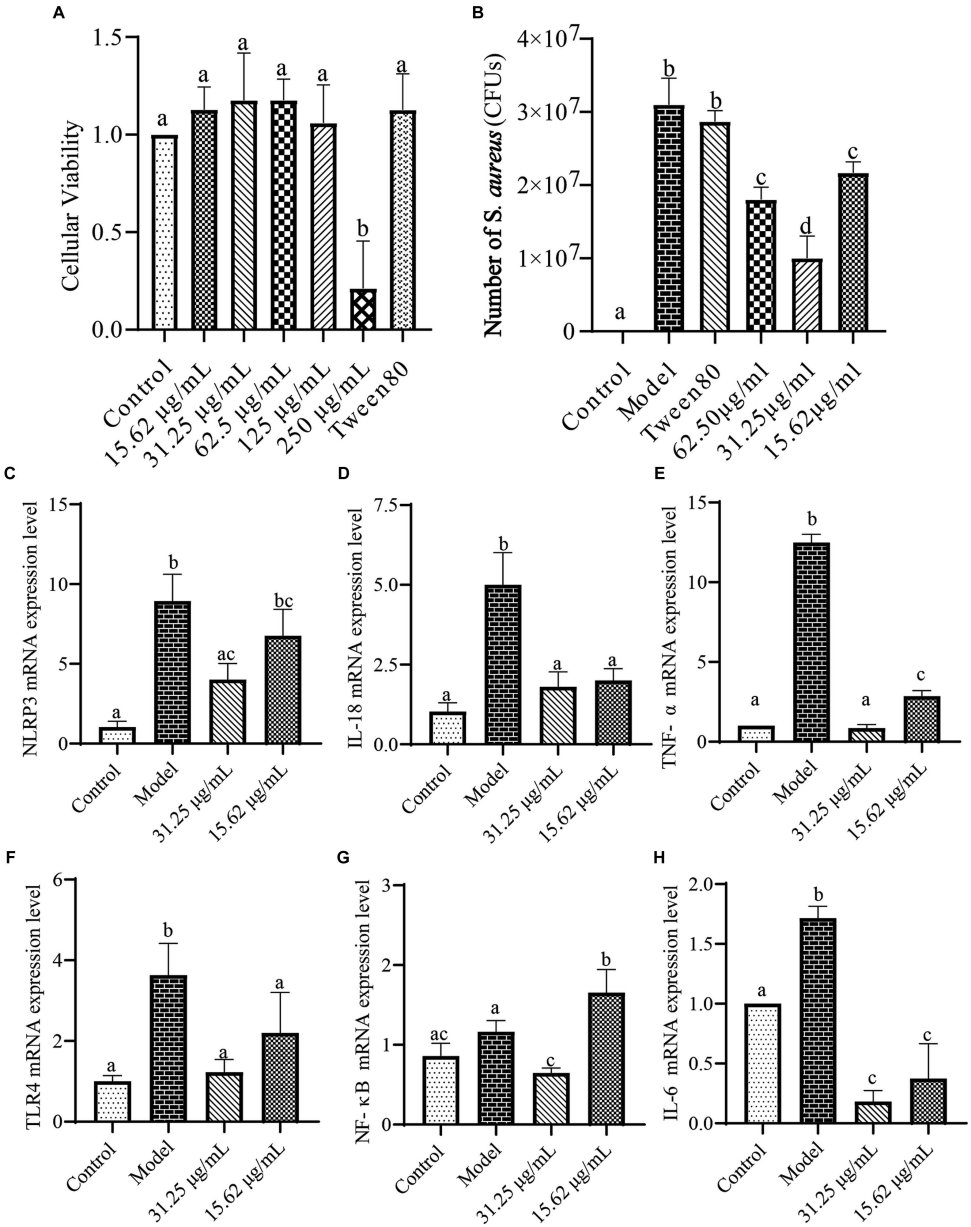
The antibacterial effects and mechanisms of WVOE on *S. aureus* infected LLC cells. **(A)** Cytotoxicity experiment of WVOE in LLC cells. After 23 h of WVOE cultivation, 100 μL of 10^8^ CFU/mL of *S. aureus* was added to each group except the control group, and the LLC cells were cultured for another 1 h. **(B)** Effects of WVOE on number of *S. aureus* in LLC cells infected with *S. aureus*. **(C–H)** Effects of WVOE on inflammatory factors gene levels in *S. aureus* infected LLC cells. Genes expressions in LLC cells were measured by qRT-PCR.

The toxicity of the WVOE on LLC cells was shown in Figure 7A. WVOE concentrations less than 125 μg/mL had no effect on LLC cell activity (*p <* 0.05). The *S. aureus* load in the model and Tween 80 groups significantly increased (*p <* 0.05, Figure 7B). Compared to the model group, the WVOE groups showed significantly reduced numbers of *S. aureus* (*p <* 0.05). Thirty-one and twenty-five hundredths μg/mL WVOE showed the greatest inhibitory effect. As shown in Figures 7C–H, the gene expression levels of *TLR4*, *NLRP3*, *NF-κB*, *IL-6*, *IL-18*, and *TNF-α* were significantly increased in the model group compared to the control group (*p <* 0.05). The expression levels of the above mentioned genes were significantly decreased in the WVOE group compared with the model group (*p <* 0.05).

## 4 Discussion

To protect its stability and mask its strong taste, volatile oil from wampee leaves was emulsified. Tween 80 is a hypotoxic emulsifier with no adverse effects (Sedaghat Doost et al., 2020). Moreover, a high emulsifier concentration reduces the turbidity point of an emulsion and reduces its stability. Based on the results of emulsion stability experiments, this study determined that the final proportion of the emulsifier was 5%. The emulsion prepared with 5% emulsifier and 5% volatile oil was relatively stable, and there was no precipitation, stratification, or flocculation under high-temperature conditions. WVOE preparations have great significance for improving the planting income of fruit farmers and the findings of this study provide a scientific basis for further research on the medicinal value of wampee leaves. We found that WVOE was more effective against *S. aureus* than *S. typhimurium*. Previous studies have shown that the MIC of an ethanol extract of wampee leaf against *S. aureus* is less than 4 mg/mL (Zhao et al., 2009). The paper diffusion method has been used to determine that the minimum inhibitory dose of volatile oil from the nutlets of *Clausena anisumolens* on *S. aureus* is 0.35 mg (Su et al., 2011). In this study, the MIC of the WVOE was 312.5 μg/mL, which was lower than the MIC of the ethanol-extracted components of wampee leaves and the bacteriostatic effect was better.

To further investigate the antimicrobial mechanism of WVOE, we used a time-dependent antibacterial curve and scanning electron microscopy. The results showed that WVOE had no significant effect on *S. typhimurium*. Therefore, we suspect that it has little effect on the intestinal flora (Deng et al., 2023). But had a good inhibitory effect on *S. aureus*. The *S. aureus* cells were lysed and died after 24 h the MBC of WVOE. Gram-negative bacteria have a cell wall outer membrane composed of lipopolysaccharides, but gram-positive bacteria do not. This cell wall structure effectively protects gram-negative bacteria from invasion (Silhavy et al., 2010). The difference in cell wall structure may explain why the WVOE had different effects on the two types of bacteria.

For *S. aureus*, biofilms are key to evading immune mechanisms. Biofilm production and aggregation are the main mechanisms through which *S. aureus* adapts to its environment. When *S. aureus* invades the human body, it accumulates quickly (Archer et al., 2011; Lister and Horswill, 2014). At this time, the biofilm can replace the bacteria and is more vulnerable to attack by the immune system and antibiotics, which greatly reduces the death of *S. aureus*. Thus, biofilms enhance the resistance of *S. aureus* to phagocytosis and antibacterial agents (Chen et al., 2021; Zhang et al., 2021). Mugwort essential oil inhibits the formation of bacterial biofilms and destroys bacterial cell walls and membranes, leading to bacterial death (Duan et al., 2015). The MIC of ginger essential oil against *S. aureus* is 1 mg/mL, and it exerts its antibacterial effects by damaging the bacterial cell membrane (Wang et al., 2020). Experimental evidence for the effects of wampee leaf volatile oil on bacteria is similar to the evidence for the effects of ginger essential oil, which may be because these volatile oils have similar compositions. We found that the WVOE inhibited the formation of *S. aureus* biofilms, which greatly increased the possibility of *S. aureus* being discovered and cleared from the body. The results indicate that WVOE itself or as an adjuvant of antibiotics, may be effective at preventing and treating *S. aureus* infection.

We further investigated the effect of WVOE on *S. aureus* by measuring the bacterial number using flow cytometry. After treatment with 1/2, 1, and 4 × MIC of WVOE, the proportion of live bacteria was 84.6%, 14.5%, and 12.8%, respectively, which was lower than the proportion in the control group (97.2%). In addition, the total number of *S. aureus* cells was much lower after treatment with 4× MIC of WVOE than that in the control group. This result further confirmed that the antibacterial activity of WVOE against *S. aureus* was dose-dependent. Moreover, transmission electron microscopy revealed that MIC and MBC of WVOE treatment both significantly induced cavities, cell wall dissipation, and the inhibition of bacterial division and proliferation in *S. aureus*.

Wampee leaf volatile oil relieves cough and asthma (Huang et al., 2015). There has no research on the antibacterial mechanism of wampee leaf volatile oil against *S. aureus*. To evaluate the bacteriostatic effects of WVOE on *S. aureus* and its related mechanisms, we established a model of *S. aureus*-infected LLC cells *in vitro*. Cell death and floating were observed with prolonged *S. aureus* infection from 1 to 2 h, resulting in abnormal experimental results for subsequent bacterial infections and further experiments. Therefore, 1 h infection of *S. aureus* was selected as the model time. Using this model, we found that the WVOE significantly reduced the number of *S. aureus* in *S. aureus*-infected LLC cells. The TLR4-NF-κB pathway is a classic inflammatory pathway activated after infection with *S. aureus* and it can cause an inflammatory response. *Schizonepeta* volatile oil has been shown to inhibit the NLRP3 and NF-κB pathways to alleviate lipopolysaccharide-induced lung inflammation in mice (Lyu et al., 2019). Mixed volatile oils of mint, eucalyptus, and spruce can alleviate the inflammatory response by inhibiting the TLR4/MyD88 signaling pathway and activating NLRP3 inflammasomes (Wang et al., 2017). In this study, *S. aureus* infection obviously increased the gene expression levels of *NLRP3*, *IL-18*, *TNF-α*, *IL-6*, *TLR4* and *NF-κB* in LLC cells, and these gene expression levels were significantly decreased after WVOE administration. Therefore, we speculate that WVOE may inhibit the TLR4-NF-κB-NLRP3 pathway to alleviate the inflammatory response triggered by *S. aureus* infection.

## 5 Conclusion

In conclusion, the WVOE significantly inhibited *S. aureus* growth and inflammation by inhibiting the TLR4-NF-κB-NLRP3 pathway in *S. aureus*-infected LLC cells. Owing to its bacteriostatic effect on *S. aureus*, it is expected to be developed on a large scale to prevent and control *S. aureus* infection.

## Data availability statement

The raw data supporting the conclusions of this article will be made available by the authors, without undue reservation.

## Ethics statement

Ethical approval was not required for the studies on animals in accordance with the local legislation and institutional requirements because only commercially available established cell lines were used.

## Author contributions

Y-NG: Writing – original draft, Methodology, Investigation. K-RH: Writing – review & editing, Methodology, Investigation. S-SL: Writing – review & editing, Methodology, Investigation. R-WM: Writing – review & editing, Supervision, Formal analysis. M-HL: Writing – review & editing, Supervision, Formal analysis. Y-MH: Writing – review & editing, Funding acquisition, Conceptualization. L-PT: Writing – review & editing, Investigation, Conceptualization.
