# Corrigendum: The effect and mechanism of volatile oil emulsion from leaves of *Clausena lansium* (Lour.) Skeels on *Staphylococcus aureus in vitro*

**DOI:** 10.3389/fmicb.2025.1575361

**Published:** 2025-02-25

**Authors:** Yan-Na Guo, Ke-Ren He, Shao-Shan Liang, Rui-Wei Mou, Meng-Han Lu, Yong-Ming He, Lu-Ping Tang

**Affiliations:** ^1^School of Life Science and Engineering, Foshan University, Foshan, China; ^2^Department of Biomedical Sciences, City University of Hong Kong, Hong Kong, China

**Keywords:** leaves of *Clausena lansium* (Lour.) Skeels, volatile oil, emulsion, *Staphylococus aureus*, *Salmonella typhimurium*

In the published article, there was an error in affiliation 2. Instead of “Department of Biomedical Sciences, University of Hong Kong, Kowloon, Hong Kong SAR, China,” it should be “Department of Biomedical Sciences, City University of Hong Kong, Hong Kong, China.”

The authors apologize for this error and state that this does not change the scientific conclusions of the article in any way. The original article has been updated.

